# Supplementary material for: Are carbon emissions trading and green financial instruments synergistic? -Comprehensive quantitative research based on content analysis
Source: PLoS One. 2024 Mar 7;19(3):e0298601. doi: 10.1371/journal.pone.0298601 (PMC10919601; doi:10.1371/journal.pone.0298601)
Supplement: S1 Data — (ZIP) [file pone.0298601.s001.zip › Data/地级市Z_F工作报告.zip!/╜¡╦╒/╠⌐╓▌╩╨/└┤╫╘╣1⁄2╓┌║┼í╛┬φ┐╦╩2╛▌═°í┐_2010.docx]

各位代表：

现在，我代表市人民政府向大会作工作报告，请予审议，并请市政协委员和其他列席人员提出意见。

一、2009年工作回顾

过去的一年是极不平凡的一年。面对国际金融危机给经济社会发展带来的前所未有的困难和挑战，我们在中共泰州市委领导下，深入贯彻落实科学发展观，紧紧依靠全市人民，提振信心、攻坚克难，扎实工作、拼搏进取，较好地完成了市三届人大二次会议确定的目标任务。

着力化解金融危机影响，国民经济保持平稳较快发展。面对全球性金融危机，及时制定和采取一系列保增长促发展的政策措施，促进经济全面回升。预计全年实现地区生产总值1650亿元，可比增长14%。其中，第一产业增加值125亿元，增长4.8%；第二产业增加值945亿元，增长14%；服务业增加值580亿元，增长14.8%。预计全市财政总收入343.8亿元，一般预算收入138.6亿元，分别增长31.1%、37.1%，增幅分别列全省第4位、第2位；市区财税增速进一步加快，比重明显提高。全社会固定资产投资突破千亿元，达1166.2亿元，增长29.6%。继续实施“5218工程”，全市粮食总产达310.2万吨，连续6年创新高，新增高效农业面积30万亩。加快推进农业产业化、市场化进程，新增龙头企业10家、农村三大合作组织314家，农业利用“三资”40亿元。强化优质农产品基地建设，新增国家级农业标准化示范区1个、国家级无公害农产品、绿色食品和有机食品共50个。泰兴农产品加工园被授予国家级农产品加工创业基地称号。建立市级农业巨灾风险准备金，高效设施农业保险覆盖面进一步扩大。扎实抓好绿化造林工作，新增造林面积12.3万亩，森林覆盖率达17.5%。全力以赴抓运行监控、结构优化、项目实施和要素协调，工业经济回升向好。全市规模以上工业实现产值3723亿元，增加值突破千亿元；预计实现利税410亿元、利润232亿元，分别增长45%、55%，增幅均居全省前列。组织实施产业振兴计划，着力推动产业转型升级。生物医药技术、船舶及配套、不锈钢制品等产业基地被授予江苏省首批特色产业基地称号。引导企业抢抓“谷底投入”机遇，加大项目开发实施力度。扬子江地佐幸注射液、兴达子午轮胎钢帘线、新源电工高压绝缘纸板等项目列入国家重点产业振兴和技术改造投资计划，安泰动力、申视塑料等一批高新项目竣工投产，中海油润滑油等一批重、特大项目成功获批。全年工业技改财务发生数737.6亿元，实施亿元以上重大技改项目145个，比上年增加25个。开展政银企融资洽谈活动，加强信用担保体系建设，设立企业应急周转金财政专户，力保企业资金链不断。积极实施品牌战略，新增中国驰名商标3个，靖江、泰兴、姜堰跻身中国商标百强县。春兰股份复牌上市。大力发展民营经济，新增私营企业7472家、个体工商户2.9万户，私营个体经济注册资本总额突破千亿元。建筑业发展势头良好，总产值达1100亿元，增长15.3%。江苏一建完成改制，江苏骏龙建设等6家公司晋升为一级资质企业。深入推进“833工程”，服务业发展速度进一步加快，占GDP的比重比上年提高1个百分点。完善政策措施，全市140家企业实现工贸分离。积极落实各项刺激消费政策，发放家电、汽车摩托车下乡补贴，减免购房契税，消费需求进一步扩大。全市社会消费品零售总额469.9亿元，增长18.7%。举办中国泰州国际旅游节，成功承办省第六届园博会，全年接待游客突破1000万人次，旅游业总收入突破100亿元。泰州华侨城一期顺利实施。金融业加快发展。浦发银行和长江商业银行泰州分行获准筹建，苏中首家村镇银行-靖江润丰村镇银行开业。年末全市各项存款余额1916.3亿元，比年初增长35.2%；贷款余额1205亿元，增长50.3%，增幅居全省第1位，其中当年新增贷款403.4亿元；全市发放小额担保贷款1.76亿元，规模居全省首位。

大力实施开放、创新双轮驱动，发展动力进一步增强。更加注重以开放促进创新、以创新带动开放，努力为经济社会发展提供强大引擎。精心组织境内外招商活动，台资、央企重大项目招引取得新突破，总投资10亿美元的纬创资通项目、总投资22亿元的中农发动物疫苗项目成功落户并开工建设。全年注册协议外资27亿美元，实际利用外资10.5亿美元，完成自营出口42.2亿美元，新签外经合同额3.85亿美元，实现外经营业额4.5亿美元。举全市之力推进医药城建设，医药园区升格为国家级医药高新技术开发区。全球最大的疫苗企业葛兰素史克等200个产业项目签约落户，150家公司注册成立，美时医疗等48家企业建成投产，分子平台、中试二期等研发平台建成运营，金迪克、疫苗中心等重点工程加快建设。省级开发区产业承载能力进一步增强，出口加工区建设有力推进。跨江联动开发取得新成效，江阴-靖江工业园区实现营业收入308亿元，增长69.3%。沿江基础设施建设加快推进，新增万吨级码头9个，靖江港区4个万吨级码头实现对外开放，高港永安港区二期顺利实施。全市港口货物吞吐量突破1亿吨。海关、国检、海事、边检等口岸查验机构为开放型经济发展作出新贡献。外事、侨务和港澳台事务工作取得新成绩，与美国威廉斯波特市结成国际友好交流城市。我市被台湾电电公会评为“值得推荐城市”，与台湾工业总会、制药同业公会签订长期战略合作协议。积极推动创新型经济加快发展。全年高新技术产业产值突破千亿元，达1137.5亿元。首批16家市级企业院士工作站成立，中科院泰州中心新入驻4家研发分中心，省级科技孵化器新增3家。春兰动力镍氢电源系统入选国家首批自主创新产品，泰州供电网无功电压优化运行集中控制系统等3个项目获得国家科技进步奖二等奖，9家企业成为全省首批创新型企业，我市成为全省唯一的国家知识产权工作示范市，并再次被评为国家科技进步先进市。成功举办第七届科技洽谈会。

强力推进中心城市建设，城乡面貌发生较大变化。2009年是城建项目最多、体量最大、投资最强的一年。紧紧抓住国家扩内需、保增长政策机遇，强化资金筹措，推进阳光拆迁，加快项目实施，全年市区城建投资达120亿元。“十大重点工程”全面实施，园博园按期开园，泰九路改造基本完工，稻河古街区改造、凤城河风景区三水湾建设、南通路改造、328国道泰姜段改线有序推进，泰州博物馆、图书馆、美术馆开工建设；鼓楼南路、海陵南路和扬子江路南延等一批路桥工程如期竣工，新老城区交叉道口改造24处，鼓楼路、税东街沿街小区环境整治基本结束。组织开展全国文明城市创建工作先进市、国家历史文化名城、国家级节水型社会建设试点市、国家生态市创建活动，中国宜居城市创建达标，国家园林城市创建通过专家组现场考核和遥感测试。数字化城管平台建设取得新成效，我市获得中国城市管理进步奖。各市加大城建力度，城区形象得到进一步提升。靖江滨江新城核心区加快建设，泰兴北部片区改造力度加大，姜堰创建国家卫生城市通过省级考核，兴化历史文化建筑保护和修缮成效明显。继续推进全面小康“十百千”工程，新创市级全面小康先行村250个；认真办好农村新“5+1”实事，新（改）建农村公路800公里，改造农村桥梁460座，改厕7.7万座，疏浚河道3923万方，泰兴、姜堰、海陵农村河道疏浚通过省级验收。城乡基础设施进一步完善，泰州长江大桥及北接线建设进展顺利，全长76公里的兴泰公路改（扩）建工程提前建成通车，宁启铁路复线及电气化改造、引江河疏港大道等重点项目相继开工建设；周山河和老通扬运河整治一期如期完成，各市城区防洪工程加快推进；500千伏凤城变等一批重点电力设施建成投运，邮政、通信等基础设施建设取得新进展。

努力促进社会全面进步，各项社会事业加快发展。贯彻教育优先方针，稳步推进区域教育现代化创建，姜堰、海陵通过省级验收；重视义务教育均衡发展，着力提升基础教育教学质量，全市高考成绩继续位居全省前列；加快高等教育发展步伐，泰州大学筹建工作取得积极进展，南京医科大学康达学院等3所高校药城校区启动建设；调整和完善市区中小学中长期布局规划。加快文化泰州建设，《泰州市历史文化名城保护规划》修编完成，非物质文化遗产名录体系建立，12部古籍入选国家级古籍保护名录；泰州大剧院建成运营，海纪馆重新布展开馆，省泰中旧址保护性改造一期、北山寺大殿和岳武穆祠修缮进展顺利，市歌舞剧院组建；隆重举行庆祝建国60周年、纪念改革开放30周年、纪念海军诞生60周年等系列文化和庆祝活动。全市有线电视用户总量突破120万户，市区有线电视数字化转换率达95%。举办第三届少儿艺术节、第五届全民健身节和老年艺术节，承办全国乒乓球超级联赛、全国男子排球联赛。在第十一届全运会上，我市运动员获得3金3银1铜的历史最好成绩。兴化被授予国际象棋之乡称号。强化基层卫生资源布局调整和卫生服务网络建设，全市建成省级示范卫生院10个、标准化卫生院88个，市区建成社区卫生服务中心26个、卫生服务站80个；加强卫生应急能力建设，有效应对甲型H1N1流感疫情。人口出生性别比例保持在正常值范围内，计生优质服务体系实现全覆盖，我市被列为全国人口和计划生育队伍职业化建设试点市。加强生态环境保护，秸秆烟害防控、企业违法排污整治等专项整治行动取得新成效。兴化戴南中日合作农村分散型污水处理示范项目如期建成。组织开展“大爱倾城、情满泰州”等慈善活动，市慈善资金规模扩大到6000万元。完成第二次全国经济普查，并获国家先进集体称号。史志档案、社会科学、新闻、物价、统计、气象、防震和民族宗教、妇女儿童等工作继续加强，质监、药监、工商管理为地方经济发展作出了新贡献，国防教育、国防动员、民兵预备役、双拥工作和人民防空事业取得新成绩。

全力保障和改善民生，人民生活水平有了新的提高。坚持把保民生作为保增长的出发点和落脚点，认真为民办好实事，努力让改革发展成果更多地惠及民众。预计全年城镇居民人均可支配收入19090元，农民人均纯收入8220元，分别增长11%、12%；城乡居民储蓄存款余额突破千亿元，达1002亿元，增长21%。积极创建国家级创业型城市，大力实施创业带动就业行动计划，鼓励和支持返乡农民工、高校毕业生以及失业人员创业，全年实施各类培训40万人次，净增城镇就业人员5.4万人、失业人员再就业2.2万人、农民创业5.35万人，城镇登记失业率控制在3%以内。出台城乡居民社会养老保险实施办法，进一步扩大社会保障受益面。降低市区职工医疗保险起付段标准，完善门诊统筹和二次救助办法，建立退休人员个人账户与养老金同步增长机制，在全省率先启动异地就医实时结算试点；提高新型农村合作医疗住院报销比例和补偿封顶线标准，新增参保人员5万人，参保率达98%。高度重视社会困难群体生活，提高城乡居民最低生活保障标准、退职老职工定补标准，及时发放老复员军人和老残疾军人遗孀生活补助，与1.6万名农村重点优抚对象签订住院医保协议，对市区939户低保家庭实行免费医保，对18809名重残无业人员发放生活救助金，基本实现市区有劳动能力并有就业意愿的残疾人“零待业”。市社会福利中心、市救助管理站、市残疾人综合服务中心建成启用。千方百计保障困难家庭住房需求，全市新建经济适用房58.2万平方米，市区新建经济适用房30万平方米，落实租售并举保障房源350套、廉租房源272套。积极发展市区公共交通，新开辟线路7条，火车站公交停车场基本建成，实现公交股权回购。区域供水工程扎实推进，农村饮用水安全工程新增受益人口45万人。抓好农村扶贫帮困工作，新增脱贫人口5.7万人。高度重视食品、药品安全，放心消费工作取得新成效。群众性精神文明创建活动深入开展，组织泰州市“突出贡献人物”评选，定期开展“百姓系列”市民素质教育活动。“百姓信服的好法官”陈燕萍成为全国重大典型。

致力加强自身建设，施政能力和水平继续提升。注重研究和分析宏观经济形势，抢抓积极财政政策和适度宽松货币政策机遇，适时出台政策性措施、指导性意见，着力化解发展中遇到的矛盾和制约。大力推进政府管理、服务创新，有序推进新一轮政府机构改革，进一步理顺和完善市区行政、财政管理体制，取消和停止行政事业性收费、政府性基金46项；深入推进“三服务”活动，完善重大项目推进机制，政府效能进一步提高。始终坚持依法行政，及时清理政府规范性文件，开展行政复议委员会试点。自觉接受市人大及其常委会的法律监督、工作监督和市政协的民主监督，重视和发挥各民主党派、工商联、无党派人士和各人民团体的参政议政作用；认真办理人大代表建议和政协提案，综合满意率达99.8%。加强基层民主法制建设和社区建设，“村民直评村官”做法得到推广，建成国家级民主法治示范村5个、全国和谐社区示范区1个。组织开展“信访积案化解年”、“大接访、大下访、大排查、大调处”等活动，各类矛盾纠纷和信访案件得到妥善处置。推进“平安泰州”建设，组织开展打击“两抢”、传销、电信短信诈骗和假币犯罪等专项行动，圆满完成大型活动安全保卫工作。社会公众安全感认可度达98.6%。高港区被评为全国平安建设先进区。建立健全各类应急预案，完善应急体制和机制，应对突发事件能力不断增强。苏中区域消防中心一期竣工。扎实推进对口援建工作，援建四川绵竹拱星镇项目全面推进，部分工程竣工交付使用。进一步深化政务公开，建成行政权力网上公开透明运行系统；着力办好政府网站，丰富和完善在线访谈等栏目，“网上政府”建设取得新成效。认真接处市长公开电话、市长信箱和行风热线，及时为群众解决实际困难和问题。落实廉政建设和反腐败工作责任制，出台公开监察新增投资项目实施意见，加大执纪执法和纠风治乱工作力度。机关公有资产管理进一步加强，节约型机关建设取得积极进展。

各位代表，过去一年政府各项工作取得的成绩，是在中共泰州市委的正确领导下，在市人大、市政协的监督和支持下，全市人民团结拼搏、共同努力的结果。在此，我代表市人民政府，向辛勤工作在全市各行各业的广大干部群众，向给予政府工作热情支持和有力监督的全体人大代表、政协委员，向市各民主党派、工商联、各人民团体和各界人士，向各驻泰机构以及驻泰部队指战员、武警官兵和公安干警，向所有关心支持泰州现代化建设的海内外朋友，表示衷心的感谢！

在充分肯定成绩的同时，我们也清醒地看到，全市经济社会发展中还存在一些问题和不足，主要是：经济回升的基础还不稳固，部分企业运营较为困难；转型升级的任务还非常艰巨，经济结构偏重、发展方式粗放、创新能力不强等都不同程度地存在；经济国际化水平有待进一步提高，利用外资规模还不够大，重大储备项目还相对较少，外贸出口增速回落；中心城市功能不够完善，集聚和辐射带动作用还不够强，社会建设、文化建设也存在不少薄弱环节；改善民生面临一些新的问题，就业形势依然严峻，农民持续增收难度还较大；政府自身建设有待进一步加强，尤其需要加快提升服务发展的能力和水平、切实提高执行力和落实力。对上述问题，我们一定高度重视，采取切实有效措施，在今后的工作中努力克服和解决。

二、2010年工作任务

各位代表，今年是实施“十一五”规划的最后一年，也是确保经济社会发展“三年再来一个大变化”的决战冲刺之年。做好今年的政府工作，具有十分重要的意义。综合分析国内外形势，总体上继续向好，但不确定、不可预料的因素依然较多。我们要辨证分析和正确把握发展形势，坚定信心、克难奋进，努力在应对挑战中抢抓机遇，在开拓进取中增创优势，确保经济社会平稳较快发展，加快走出一条具有泰州特色的创新发展之路。

今年政府工作的总体要求是：全面贯彻党的十七届四中全会、中央和省经济工作会议精神，认真落实市委三届七次、八次全会决策部署，以科学发展观统揽全局，紧紧围绕“三年再来一个大变化”，坚持开放创新双轮驱动，进一步加快经济转型升级、增强中心城市功能、推进城乡统筹发展、提高人民生活水平，奋力开创泰州科学发展新局面。

今年全市国民经济和社会发展主要调控目标为：地区生产总值可比增长12%；财政总收入增长15%，一般预算收入增长15%；全社会固定资产投资增长20%；社会消费品零售总额增长16%；自营出口额增长5%；实际利用外资增长10%；城镇居民人均可支配收入增长10%，农民人均纯收入增长9%；居民消费价格涨幅不高于全省平均水平；城镇登记失业率控制在3.5%以内；研发投入占地区生产总值比重1.8%，高新技术产业产值占规模以上工业比重30%以上；万元GDP能耗下降5%。

今年将重点做好五个方面的工作：

1. 推进转型升级，力促经济又好又快发展。在巩固和发展经济回升向好势头的同时，把调结构、促转型摆上突出位置，努力在转变经济发展方式、促进区域共同发展上取得更大进展。

大力发展新兴产业。加快医药产业发展，突出抓好医药高新区领军人才、研发机构引进和科技成果产业化，加快龙头型产业项目、研发制造支撑平台和基础设施建设，力争早日实现“双千目标”。加快电子信息产业发展，着力抓好纬创资通项目实施，积极引进相关企业和高端产品，打造电子信息产业集群。加快新能源产业发展，重点实施一批太阳能发电系统及配套产品、核电风电配套产品和新型电池等重点项目，形成新的重要增长点。科学规划和加快培育新材料、新型环保设备、智能电网等产业，拉长产业链，促进规模化。高度重视低碳经济、绿色经济和循环经济发展，坚决淘汰落后产能，积极推广高效节能技术和产品，努力完成“十一五”节能减排任务。大力发展现代物流、金融保险、服务外包、软件开发等生产性服务业，为新兴产业发展提供有力支持。年内新增省级现代服务业集聚区1-2家，力争把医药高新区打造成国家级服务外包示范区。

切实增强创新能力。坚持开放式创新与自主创新并举，用高新技术和先进适用技术加快改造、提升传统支柱产业，不断增强产业竞争力。鼓励和支持企业引进科技成果、开展技术合作、推进创新体系建设，努力突破一批关键技术、转化一批重大科技成果、培育壮大一批创新型龙头企业。年内，全市重点建设100个产学研联合体，跟踪实施20个重大科技专项，上争和组织实施国家、省级各类科技项目100个以上，引进一批高层次创新创业人才，市及各市（区）均成立风险投资公司或创业投资公司。全年高新技术产业产值力争突破1400亿元。认真落实《关于进一步促进中小企业发展的若干意见》，切实改善创新创业环境，培育一批创新型中小企业；实施百企上市培育计划，新增一批企业进入上市辅导期或实现上市。

加快发展县域经济。坚持分类指导，强化服务支持，提升县域经济整体发展水平，努力形成各具特色、充满活力、奋勇争先的生动局面。引导各市发挥自身优势，找准目标定位，加快转型升级，进一步做大做强主导产业和特色产业。促进工业集中区集约、集群发展，突出抓好项目招引和产业集聚，着力打造一批有影响、有特色的经济板块。全力支持各市上争项目，高效做好协调、服务工作，积极帮助化解发展难题。认真落实帮扶政策措施，推动黄桥老区和里下河地区加快发展。

着力壮大市区经济。按照市区经济率先转型升级、加快发展的要求，坚持一手抓总量扩张、一手抓质态提升，推动市区经济发展壮大，切实提高占全市经济比重。调整优化市区产业发展格局，进一步明晰产业定位和发展方向。强化市区新兴产业功能区建设，更好地集聚高新项目和高端人才。完善市区管理体制机制，合理划分事权和财权，激发市区发展活力。进一步增强对市区产业发展的政策支撑，不断提升市区经济综合竞争力。

2. 抓好“三农”工作，推动农村经济社会全面发展。坚持以提高农民收入为核心，大力发展农村经济，加快推进新农村建设。

积极发展现代农业。继续实施“5218工程”，推进农业适度规模经营，着力打造一批特色农业园区和规模基地。开展高产增效创建示范活动，新增万亩粮油高产增效示范片30个、高效农业面积21万亩。整合农业科技推广资源，加快构建种养业良种繁育体系，促进先进适用技术落地应用。开展“农产品质量建设推进年”活动，建设优质农产品生产基地。加大高效设施农业保险推进力度，规模种养业项目保险实现全覆盖。抓好以绿色通道建设为重点的林业绿化工作，新增造林面积12万亩以上。完善动植物疫病防控体系，保证农产品质量安全。

继续推进新农村建设。认真办好农村新一轮实事，不断改善农村生产生活条件。抓好农村道路建设，新（改）建农村公路500公里。按照“全国亿万农民健康促进行动”部署，广泛开展农村健康知识普及，建成1-2个省级示范县（市）。继续实施农村饮水安全工程，新增受益人口18.8万人。加强农村环境综合整治，加快秸秆能源化利用，整治疏浚农村河道，推进农村户厕无害化改造，完善城乡垃圾收运、处理体系。深入开展环境优美乡镇争创活动，全面完成“十百千”工程建设任务，新创成国家级环境优美乡镇3个、市级全面小康先行村200个以上。加大扶贫帮困力度，确保完成脱贫攻坚目标任务。

努力促进农民增收。围绕提高农民种养业收入，积极推广种养新模式、新品种；大力发展订单农业，支持农业产业化龙头企业加快发展；继续组织好农产品营销工作，进一步扩大市场份额，促进农民增收。围绕提高农民经营性收入，大力发展农村三大合作组织，年内新增300家；强化农民创业培训，扩大农村小额贷款规模，扶持农民创业。围绕提高农民工资性收入，适应重大项目建设用工需求，抓好专业技能培训，促进农民本地转移就业。全年组织农民实用技术培训15万人、创业培训8000人，新增创业农民5万人、创业带动就业15万人。

3. 扩大对外开放，提高开放型经济发展水平。认真研究新形势下境外资本转移规律、贸易发展动态，努力在更广领域、更高层次上参与国际竞争与合作。

更大力度推进招商引资。进一步加大行政推动力度，切实提高招商活动实效。继续突出抓好台资和央企招引，放大纬创资通落户的集聚效应，组织高密度的赴台招商，加快打造台商投资集聚区；强化央企投资信息追踪，组织好各类推介活动，力争获得更多的央企研发机构和产业基地布点。着力加强产业链招商，重点围绕医药高新区和纬创资通项目建设，强化上下游产业和关联产业项目的招引，加快形成医药和IT产业集群。加强引资载体建设，引导医药高新区和各省级开发区做好新一轮规划修编，培育特色园区。深入推进沿江开发，积极做好沿海开发策应工作。

保持外贸出口稳定增长。抢抓全球经济逐步复苏有利时机，转变外贸增长方式，积极发展对外贸易。进一步优化出口结构，力争机电和高新技术产品的占比达65%以上；健全自主出口品牌促进机制，培大育强骨干企业，新增一批出口超3000万美元企业。加快出口产业集聚区建设，力争获批国家级出口加工区。深化出口企业服务，落实国家信用保险政策措施，打造“贸银信”合作平台；支持企业参加各类会展，积极开拓国际市场。指导和服务企业有效应对贸易摩擦。

加快“走出去”步伐。支持有实力的企业到境外投资、兴办实业，在更高层次上利用“两种资源”、“两个市场”，提升国际竞争力。鼓励有条件的企业与跨国公司、知名企业高端合作，在战略性合作并购重组中实现高位嫁接。积极推进重点企业开展境外资本运营，实现境外上市。支持企业承揽总承包业务，着力提高对外承包工程和劳务合作水平；规范外派劳务，加强境外劳务纠纷和突发事件的应急处置工作。重视和加强外事、侨务工作，促进对外交流合作。

4. 加快城市建设，提升中心城市集聚和辐射能力。按照南进东扩、组团发展、功能互补、生态宜居的要求，坚持市区一体、多点并进，强力推进大项目建设，加快拉开大城市框架，全面掀起大推进热潮。

进一步优化城市布局。强化规划引领，注重形态设计，完善空间架构。完成新一轮城市总体规划修编和各类分区规划、控制性详细规划编制，组织进行重要区域和关键节点的城市设计。加快城市交通骨架体系建设，启动231省道泰高路改线和站前路建设，推进引江河疏港大道、泰州大桥北接线工程，建成328国道泰姜段改线。统筹推进南部新城区规划建设，重点实施海军大道东延和东风路、鼓楼路、海陵路南延等“四路七桥”工程，启动建设新区高级中学、市人民医院新院，建成医药高新区会展中心。有序推进北部老城区建设，突出抓好东、西大门和青年路两侧改造，实施迎春路、迎宾路东延等老城区道路畅通工程，着力解决“断头路”问题。积极推进滨江港城区建设，实施春港路、迎江路、环港大道等路桥项目，加快打造泰州“外滩”。

进一步完善城市功能。围绕加快集聚人气、商气，着力繁荣坡子街商圈，规划建设周山河街区城市商贸中心。积极引进新型流通业态、大型商贸企业，实施济川路城市综合体、华钜金海广场、大润发超市、红星美凯龙等重点项目，推进市区农贸市场建设和升级改造，建设东风路农产品批发等特色市场。加快博物馆、图书馆、美术馆、文化馆、规划展示馆、青少年活动中心、未成年人社会实践基地等场馆设施建设，启动新体育中心规划设计；继续推进市区长江水通乡达镇和燃气进老小区，确保城南污水处理厂二期竣工、市区污水管网实现全覆盖；大力发展城市公交，加大投入、强化管理，突出抓好公交车辆更新、线路优化和公交站场建设，确保市区公交服务水平得到显著提升。继续完善数字化城管二期工程，综合整治无物管小区环境，切实加强公共停车场建设。

进一步提升城市品位。坚持走内涵发展之路，加快塑造和展现城市个性、品质。着力彰显历史文化底蕴，扎实抓好历史遗存保护、修复，改造复兴稻河古街区，启动建设城中、渔行等历史文化街区，完成北山寺、岳武穆祠修缮和俞氏等古民居保护工程。致力打造城河文化精品，围绕“水位可亲、水体流畅、水质优良”目标，实施城市水生态环境建设工程，完成周山河、老通扬运河二期整治；加快凤城河沿河景观带建设，扩建柳敬亭公园，繁荣三水湾时尚休闲街区。大力发展旅游文化，规划建设滨江生态湿地旅游度假区，启动建设大桥生态景观公园，支持泰州华侨城建设和开发，举办第二届中国泰州国际旅游节，打造长三角旅游目的地。深入推进全国文明城市创建工作先进市创建活动，认真做好国家历史文化名城创建迎检工作，组织实施生态市建设行动计划，确保通过国家环保模范城市复查，创成国家园林城市和江苏省节水型城市。

进一步加快城乡重大基础设施建设。积极配合和服务泰州长江大桥、苏中机场、宁启铁路复线及电气化改造、江海高速泰州段、泰镇高速泰州段、宁通高速泰州段扩容等重点项目建设，规划建设泰州港核心港区，建成京泰汽车客运站。实施引江河二期、卤汀河拓浚和城郊水资源保护，深入推进区域供水工程。加快实施500千伏泰兴变扩建、220千伏洋桥变等输变电项目，确保年内建成投运。继续完善通信等基础设施。

进一步推进城乡一体化发展。统筹抓好各市城区和镇村建设，引导各市加快城区建设步伐，推动黄桥、戴南、溱潼、新桥等中心城镇加快向小城市方向发展，培育一批特色明显的中小城镇。着力抓好市区村组（社区）股份制改革和社区建设工作，加快撤镇设街、撤村设居和城中村、园中村、城郊村改造步伐。积极推进户籍制度改革，放宽城镇落户条件，引导农民逐步、有序融入城镇。继续推动城市生产要素向农村流动、基础设施向农村延伸、公共服务向农村覆盖，有效扩大城乡消费需求。

5. 保障改善民生，促进社会和谐稳定。在保增长促发展的同时，更加关注民生福祉，加强社会建设与管理，构建和谐泰州。

突出抓好就业和社会保障工作。扎实推进国家级创业型城市创建工作，确保通过国家验收；整合各类就业创业扶持政策，建立面向城乡的普惠化政策体系，行政村全部建成村级创业就业服务平台，促进民营经济加快发展；实施就业再就业援助，动态消除“零就业”家庭，全年净增城镇就业人员4万人。完善城乡居民养老保险办法，建立农村基本养老保险金、老年农民养老补贴调整机制，确保新农保实现全覆盖、参保农民达120万人；调整医疗保险政策，提高参保人员保险待遇，新型农村合作医疗保险参保率稳定在98%以上，全市人均筹资标准不低于150元、市区达180元；推进养老和工伤保险统筹，不断提高基金支付能力。进一步完善被征地农民社会保障制度，确保参保率达90%。加强劳保监察和劳动争议调处工作，维护企业职工和进城务工农民合法权益，构建和谐稳定劳动关系。促进房地产业稳定健康发展，突出抓好保障性住房建设，着力解决低收入家庭住房困难，市区新开工建设经济适用房5万平方米，新增租售并举经济适用房350套和廉租房源、公共租屋各50套。完善城乡低保分类救助和特困救助制度，对中等职业学校家庭经济困难学生和涉农专业学生免收学费，对农村义务教育公办学校寄宿生免收住宿费，对残疾人实施生活救助和就业安置。

繁荣各项社会事业。加快教育现代化步伐，靖江、泰兴和高港区域教育现代化创建通过省级验收；促进义务教育均衡发展，恢复义务教育阶段“公有民营”学校公办性质，均衡配置优质教育资源；启动全市中小学校舍安全工程，迁建市区康和学校；大力发展职业教育，增强服务地方经济发展的能力；继续推进泰州大学筹建工作。加快发展文化事业和文化产业，鼓励文艺创作，办好第二届梅兰芳艺术节等重大文化节庆活动；加大文化体制改革力度，完成市淮剧团改制工作；继续加强乡镇（街道）宣传文化中心和村文化室建设，力争实现“农家书屋”全覆盖。推进各市有线电视数字化整体转换，建设泰州地面数字电视传输平台。深化医药卫生体制改革，开展公立医院改革试点和基本药物制度试点；完善公共卫生服务体系，市区重点建设10个社区卫生服务中心、40个服务站。积极申办重大体育赛事，做好省第十七届运动会参赛工作。落实计生保障优惠政策，积极创建“全省人口协调发展先进县（市、区）”。扎实做好第六次全国人口普查工作。完善社会福利服务体系，大力发展慈善事业。加快养老服务社会化步伐，开工建设海陵区养老院、高港区养护院。继续推进人民防空与防灾一体化建设。积极发展史志档案、社会科学、新闻、物价、统计、气象、防震等事业，认真做好民族宗教和妇女儿童等工作。

加强精神文明和民主法制建设。贯彻落实《公民道德建设实施纲要》，深入实施“市民文明素质提升工程”。以文明行业（单位）、社区、村镇创建为重点，推进城乡文明和谐共建活动。组织开展“百万市民学礼仪”、“文明交通”、志愿者服务等道德实践活动，努力放大“百姓系列”、“爱心”品牌效应。大力宣传陈燕萍等先进典型，充分发挥示范引领作用。完善学校、家庭、社会“三结合”网络，进一步加强和改进未成年人思想道德建设。深入开展双拥共建活动，做好国防教育、国防动员、民兵预备役和兵员征集工作。大力推进村（居）民自治，认真组织村（居）委会换届选举。继续抓好“五五”普法，增强全体公民法律意识。加强社会管理创新，维护社会和谐稳定。完善群众利益诉求协调机制，落实信访工作领导责任制，坚持领导干部定期接访和下访，切实解决一批疑难信访案件；加强调解工作制度化、规范化建设，积极调处、化解社会矛盾纠纷；落实重大决策、重大项目社会稳定风险评估制度，从源头上预防和减少社会矛盾。开展第三轮“平安泰州”建设，深化社会治安综合治理，推进信息化、立体化社会治安防控体系建设，抓好社会闲散青少年教育、流动人口管理、刑释解教人员帮教安置工作；开展“五星级”村（社区）创建活动，综合整治城中村、城郊村等治安复杂地区，大力推进外来人口“新市民工程”。进一步强化应急机构和救援队伍建设，积极预防和妥善处置突发性公共事件。加强安全生产监督检查，严防重特大事故发生。实施食品、药品放心工程，保障人民群众生命健康安全。

三、加强政府自身建设

各位代表，科学应对当前宏观经济形势，加快推进“三年再来一个大变化”，全面完成“十一五”规划任务，都对政府工作提出了新的、更高的要求。我们将以打造实干政府、阳光政府、法治政府、廉洁政府为主线，进一步加强政府自身建设，更好地肩负起建设和谐美好新泰州的重任。

1. 着力打造实干政府。坚持大干快干、苦干实干，促进各项工作大见效、快见效。大力倡导雷厉风行的工作作风，立说立行、紧张快干，确保既定工作快速上手，工程项目强力推进，各项目标早日完成。大力倡导敢于碰硬的认真态度，知难而进、迎难而上，以改革创新的思路化解矛盾、突破困局，下决心消除影响和制约发展的体制机制性障碍，解决一批群众关注的热点难点问题。大力倡导一抓到底的执着精神，把抓落实作为政府工作的生命线，紧紧围绕市委的各项决策部署，细化、实化操作性措施和办法，一件一件地落实，一项一项地推进，切实提高执行力和落实力。

2. 着力打造阳光政府。坚持以公开为原则、不公开为例外，进一步推进政府信息公开，保障公众充分享有知情权；坚持问政于民、问需于民、问计于民，涉及群众切身利益的重大决策和相关措施出台前，主动听取人大代表、政协委员和群众的意见，保障公众行使参与权；坚持将权力运行全过程置于“阳光”之下，深化行政权力网上公开透明运行工作，保障公众行使监督权。把建设“网上政府”作为打造阳光政府的重要抓手，继续强化政民互动平台建设，切实办好“望海楼论坛”，认真接处市长信箱群众来信，高度重视政府新闻发布和网络舆情应对工作。

3. 着力打造法治政府。按照法定权限和程序，行使权力、履行职责。建立规范性文件科学运行和定期审查机制，积极推行行政复议集中受理，强化行政执法行为监督，加大行政问责力度，严肃查处行政不作为和乱作为。自觉接受市人大及其常委会的法律监督和工作监督，积极支持政协履行政治协商、民主监督和参政议政职能，主动听取各民主党派、工商联、无党派人士和各人民团体的意见，继续办好人大代表建议和政协提案。进一步转变政府职能，深化行政管理体制改革，完成市及市（区）政府机构改革任务。切实做好“十二五”规划纲要编制工作。

4. 着力打造廉洁政府。认真落实党风廉政建设责任制，加强教育、制度、监督并重的惩治和预防腐败体系建设，扎实推进反腐倡廉工作。深化廉政文化建设，引导公务员强化廉洁从政意识。积极构建政府投资项目监督管理制度体系，强化对重点领域、重点环节、重点项目的审计监督和行政监察，依法查办各类违纪、违法案件。深化纠风和工程建设领域专项治理工作，从严整治政风、行风。坚持艰苦奋斗、勤俭节约，压缩行政支出，把有限财力、物力用到发展经济与改善民生上，始终保持为民务实清廉的良好形象。

各位代表，泰州已经站在一个新的发展起点上，机遇与挑战并存，希望与困难同在。让我们紧密团结在以胡锦涛同志为总书记的党中央周围，在中共泰州市委的领导下，紧紧依靠全市人民，解放思想、锐意创新，抢抓机遇、拼搏进取，为全面完成今年经济社会发展和“十一五”规划各项目标任务，加快实现“三年再来一个大变化”而努力奋斗！
